# Supplementary material for: Gut Microbiota Markers and Dietary Habits Associated with Extreme Longevity in Healthy Sardinian Centenarians
Source: Nutrients. 2022 Jun 12;14(12):2436. doi: 10.3390/nu14122436 (PMC9227524; doi:10.3390/nu14122436)
Supplement: Supplementary file 1 [file nutrients-14-02436-s001.zip › nutrients-1761110-supplementary.pdf]

Gut microbiota markers and dietary habits associated with extreme longevity in healthy Sardinian centenarians

Vanessa Palmas[1], Silvia Pisanu[1], Veronica Madau[1], Emanuela Casula[1], Andrea Deledda[2], Roberto Cusano[3], Paolo Uva[4], Andrea Loviselli[2], Fernanda Velluzzi[2°], Aldo Manzin[1°*]

[1]Department of Biomedical Sciences, University of Cagliari, Cagliari, Italy
[2]Department of Medical Sciences and Public Health, University of Cagliari, Cagliari, Italy
[3]CRS4, Science and Technology Park Polaris, Piscina Manna, Pula, Cagliari, Italy
[4]IRCCS Istituto Giannina Gaslini, Genoa, Italy

°These authors contributed equally to the work

* Correspondence to: Prof. Aldo Manzin, Department of Biomedical Sciences, University of Cagliari, Microbiology and Virology Unit, Azienda Ospedaliero-Universitaria Cagliari, Strada Statale 554-bivio Sestu, I-09042 Monserrato, Cagliari (e-mail: aldo.manzin@gmail.com).

Materials and Methods

Anthropometric evaluation

The anthropometric evaluation included measurements of height (cm) and weight (kg), used for the Body Mass Index (BMI) calculation (kg/m^2). BMI was calculated by the ratio of weight in kilograms and height in meters squared: normal range (normal-weight condition) was considered 18.5-24.99 kg/m2, the range for overweight was 25-29.99 kg/m^2, for obesity class I was 30-34.99 kg/m^2, for obesity class II was 35-39.99 kg/m^2 and for obesity class III greater than or equal to 40 kg/m^2.

Lifestyle factors assessment

The MDS score, including 11 food groups, assigns an increasing score from 0 to 5, based on the monthly or weekly consumption of foods typical of the Mediterranean diet (unrefined cereals, potatoes, vegetables, fruit, legumes, fish and olive oil) and a decreasing score from 5 to 0, based on the monthly consumption of foods not included in the same dietary model (meat or derivatives, poultry, whole dairy products); for alcohol consumption, decreasing scores from 5 to 1 correspond respectively to a consumption <300 ml/day, <400 ml/day, <500 ml/day, <600 ml/day, while a score of 0 corresponds to a consumption > 700 ml/d or no consumption. The total score, obtained from the sum of the 11 individual scores (one for each food group), ranges from 0 to 55, where higher values indicate greater adherence to MD [1].

State of cognition was assessed through the MMSE questionnaire. The score ranges from 0 to 30, with higher scores corresponding to better cognitive functioning [2]. However, in the assessment of the elderly population, the score must be appropriate to the age group and level of education [3].

The ability of each subject to carry out the daily life activities independently was measured through the ADL questionnaire. If the person is unable to carry out any activity, he/she is considered functionally incapable (ADL dependent) [4].

Nutritional status in older people was measured using the MNA questionnaire. It is an 18-item questionnaire that consists of a two-step procedure: the MNA-SF (6 elements) to screening malnutrition and its risk and the complete MNA to assess nutritional status. The questionnaire includes anthropometric measurements combined with information on diet, lifestyle, medications, the presence of acute stress, dementia or depression and a self-perception of health and nutrition. It correlates with clinical indicators of nutritional status (albumin level, BMI, energy intake and vitamin status). A score ≥24 corresponds to a normal nutritional status, a score ≤ 17 identifies malnourished subjects, while subjects with a score between 17 and 23.5 are at risk of malnutrition [5,6].

The level of daily physical activity in the elderly was assessed through the PASE questionnaire. It consists of 12 elements that evaluate the duration, frequency, extent of exertion and the amount of physical activity performed over a seven-day period by individuals aged 65 and over. In particular, the questionnaire evaluates the activities typically chosen by the elderly, such as walking, recreational activities, exercise, housework, gardening and caring for others. The score (from 0 to 793) is calculated based on the frequency, duration and level of intensity of the activity compared to that of the previous week; higher scores indicate greater physical activity [7].

Table S1 Alpha diversity analysis between CENT, NON and CTLs and between CPAR and COFF

**Shannon index**	**Mean (SD)**	**overall *p***	**Bonferroni *p* (CENT *vs* NON)**
		**0.037**	**0.020**
**CENT**	2.39 (0.31)		
**NON**	2.46 (0.29)		
**CTLs**	2.32 (0.2)		
		0.398	
**CPAR**	2.40 (0.29)		
**COFF**	2.29 (0.19)		

Differences in the Shannon index between CENT, NON and CTLs, evaluated by Kruskal-Wallis test in SPSS software v.28.0.1.0 followed by Bonferroni correction for multiple comparisons. CENT= centenarian subjects, NON= nonagenarian subjects, CTLs= healthy younger controls. CPAR=centenarian parents, COFF=centenarians' offspring. Bold values denote statistical significance ($p\leq 0.05$).

Table S2 GM beta diversity analysis between CENT, NON and CTLs

**Beta divesity**		**overall *p***	**PERMANOVA pairwise *p* (CENT *vs* CTLs)**	**PERMANOVA pairwise *p* (NON *vs* CTLs)**	**PERMANOVA pairwise *p* (CENT *vs* NON)**
		**0.001**	**0.006**	**0.003**	n.s.
**CENT**					
**NON**					
**CTLs**					
**Degrees of freedom**	2				
**Sum of squares**	1.498				
**Mean of squares**	0.749				
**F**	6.074				
**R^2**	0.1201				

Differences in the beta diversity index between CENT, NON and CTLs were evaluated by Permutational Multivariate Analysis of Variance (PERMANOVA) performed in R (RVAdeMemoire package). CENT= centenarian subjects, NON= nonagenarian subjects, CTLs= healthy younger controls. Bold values denote statistical significance ($p\leq 0.05$); n.s.= not significant. Pairwise PERMANOVA test confirmed significant segregation in the comparison between LLS groups and CTLs.

Table S3 GM beta diversity analysis between CPAR and COFF

**Beta divesity**	**PERMANOVA pairwise *p***
**CPAR COFF**	1
**Degrees of freedom**	1
**Sum of squares**	0.08
**Mean of squares**	0.08
**F**	0.706
**R^2**	0.056

Differences in the beta diversity index between CPAR and COFF were evaluated by Pairwise Permutational Multivariate Analysis of Variance (Pairwise PERMANOVA) performed in R (RVAdeMemoire package). CPAR=centenarian parents, COFF=centenarians' offspring. Bold values denote statistical significance ($p \leq 0.05$).

Table S4 29 Statistically significant common differences in the relative abundance of bacterial taxa in the two classes of LLS compared to CTLs

					**Post-hoc analysis, Bonferroni method (only for significant bacteria)**						
**Phylum**	**Family**	**Genus**	**Species**	**Kruskal-Wallis *p*-value**	**Pairwise group**	**Pairwise *p*-value**	**Chi square (χ^2)**	**↓/↑**	**Mean±SD CENT**	**Mean±SD NON**	**Mean±SD CTLs**
Actinobacteria				0.0018	CENT- CTLs	0.0393	12.66	↑	8.04±10.23	9.12±9.91	3.09±3.61
					NON- CTLs	0.0039		↑			
Actinobacteria	Coriobacteriaceae			0.0003	CENT- CTLs	0.0445	16.16	↑	1.10±1.13	1.17±1.41	0.56±0.87
					NON- CTLs	0.0004		↑			
Actinobacteria	Coriobacteriaceae	*Collinsella*		0.0022	NON- CTLs	0	12.26	↑	0.66±0.73	0.77±1.28	0.00±0.00
					CENT- CTLs	0		↑			
Actinobacteria	Coriobacteriaceae	Eggerthella		0.0075	CENT- CTLs	0.0275	9.79	↑	0.10±0.22	0.05±0.05	0.03±0.05
					NON- CTLs	0.0394		↑			
Actinobacteria	Bifidobacteriaceae	*Bifodobacterium*	*B. asteroides*	0.0006	CENT- CTLs	0.0294	14.95	↑	0.11±0.18	0.11±0.14	0.02±0.03
					NON- CTLs	0.0011		↑			
Actinobacteria	Coriobacteriaceae	*Collinsella*	*C. intestinalis*	0.0048	CENT- CTLs	0.0295	10.66	↑	0.13±0.22	0.06±0.06	0.09±0.33
					NON- CTLs	0.0192		↓			
Bacteroidetes				0.0002	CENT- CTLs	0.0032	16.95	↓	24.74±24.26	25.28±17.60	43.59±21.64
					NON- CTLs	0.0019		↓			
Bacteroidetes	Bacteroidaceae			0.0007	CENT- CTLs	0.0085	14.54	↓	15.77±17.53	15.03±12.52	28.05±18.05
					NON- CTLs	0.004		↓			
Bacteroidetes	Bacteroidaceae	*Bacteroides*		0.0007	CENT- CTLs	0.0085	14.54	↓	15.77±17.53	15.03±12.52	28.05±18.05
					NON- CTLs	0.004		↓			
Bacteroidetes	Bacteroidaceae	*Bacteroides*	*B. rodentium*	0.0002	CENT- CTLs	0.0446	17.37	↓	1.82±2.47	0.95±1.11	2.65±2.36
					NON- CTLs	0.0002		↓			
Bacteroidetes	Bacteroidaceae	*Bacteroides*	*B. sartorii*	0.0002	CENT- CTLs	0.0006	17.30	↓	0.22±0.60	0.12±0.10	0.25±0.20
					NON- CTLs	0.0102		↓			
Bacteroidetes	Bacteroidaceae	*Bacteroides*	*B. xylanisolvens*	0.0005	CENT- CTLs	0.0017	15.19	↓	0.50±0.47	0.74±0.82	1.74±2.57
					NON- CTLs	0.014		↓			
Cyanobacteria	*Aphanizomenonaceae*	*Dolichospermum*		0.0019	CENT- CTLs	0.0045	12.57	↓	0.00±0.00	0.01±0.01	0.34±1.30
					NON- CTLs	0.0348		↓			
Cyanobacteria	Aphanizomenonaceae	*Dolichospermum*	*D. macrosporum*	0	CENT- CTLs	0.0005	26.32	↓	0.00±0.00	0.00±0.00	0.34±1.30
					NON- CTLs	0		↓			

Euryarchaeota				0	CENT- CTLs	0	19.43	↑	0.29±0.65	0.12±0.36	0.00±0.00
					NON- CTLs	0.0131		↑			
Euryarchaeota	Methanobacteriaceae			0	CENT- CTLs	0	40.68	↑	0.29±0.65	0.12±0.36	0.03±0.20
					NON- CTLs	0.013		↑			
Euryarchaeota	Methanobacteriaceae	*Methanobrevibacter*	*M. smithii*	0	CENT- CTLs	0	25.27	↑	0.28±0.61	0.11±0.34	0.04±0.19
					NON- CTLs	0.0116		↑			
Euryarchaeota	Methanobacteriaceae	*Methanobrevibacter*		0	CENT- CTLs	0	24.45	↑	0.29±0.65	0.12±0.36	0.04±0.20
					NON- CTLs	0.0189		↑			
Firmicutes	Thermicanaceae			0.0051	CENT- CTLs	0.0244	10.55	↑	0.11±0.21	0.14±0.23	0.03±0.06
					NON- CTLs	0.0251		↑			
Firmicutes	Lachnospiraceae	*Butyrivibrio*		0.0016	CENT- CTLs	0.0038	12.93	↓	0.02±0.02	0.02±0.03	0.12±0.34
					NON- CTLs	0.0318		↓			
Firmicutes	Bacillales_Family X_Incertae Sedis	*Thermicanus*		0.0051	CENT- CTLs	0.0244	10.55	↑	0.11±0.21	0.14±0.23	0.03±0.06
					NON- CTLs	0.0251		↑			
Firmicutes	*Lachnospiraceae*	*Butyrivibrio*	*B. proteoclasticus*	0.0016	CENT- CTLs	0.0039	12.89	↓	0.02±0.02	0.02±0.03	0.12±0.34
					NON- CTLs	0.0327		↓			
Firmicutes	Lactobacillaceae	*Lactobacillus*	*L. taiwanensis*	0.0001	CENT- CTLs	0.0313	18.86	↑	0.02±0.05	0.16±0.84	0.00±0.00
					NON- CTLs	0.0001		↑			
Proteobacteria	Alcaligenaceae			0.0003	CENT- CTLs	0.0065	16.38	↓	0.46±1.31	0.46±1.43	0.83±0.86
					NON- CTLs	0.0014		↓			
Proteobacteria	Xanthomonadaceae			0	CENT- CTLs	0.0015	45.43	↑	0.13±0.15	0.09±0.11	0.01±0.03
					NON- CTLs	0.0021		↑			
Proteobacteria	Oxalobacteraceae	Collimonas		0	CENT- CTLs	0	61.68	↓	0.00±0.00	0.00±0.00	0.32±0.54
					NON- CTLs	0		↓			
Proteobacteria	Sutterellaceae	*Sutterella*		0.0003	CENT- CTLs	0.0068	16.32	↓	0.43±1.17	0.46±1.43	0.80±0.84
					NON- CTLs	0.0015		↓			
Proteobacteria	Desulfovibrionaceae	*Desulfovibrio*	*D. piger*	0.0004	CENT- CTLs	0.0093	15.42	↑	0.14±0.36	0.20±0.72	0.03±0.09
					NON- CTLs	0.002		↑			
Synergistetes				0.0007	CENT- CTLs	0.0024	14.54	↑	0.56±0.95	0.28±0.70	0.04±0.07
					NON- CTLs	0.0154		↑			

Table shows the GM significant differences in the two classes of LLS compared to CTLs performed by Kruskal-Wallis test on R software v.3.5.2. Pairwise comparison was performed only for significant taxa, followed by Bonferroni correction for multiple comparisons. *p* equal to or less than 0.05 was considered statistically significant. CENT= centenarian subjects, NON= nonagenarian subjects, CTLs= healthy younger controls, ↓= significantly reduced in the first term of the pairwise group, ↑= significantly increased in the first term of the pairwise group.

Table S5 24 Statistically significant divergences in the relative abundance of bacterial taxa observed only from the comparison between CENT and CTLs

					**Post-hoc analysis, Bonferroni method (only for significant bacteria)**						
**Phylum**	**Family**	**Genus**	**Species**	**Kruskal-Wallis *p*-value**	**Pairwise group**	**Pairwise *p*-value**	**Chi square (χ^2)**	**↓/↑**	**Mean±SD CENT**	**Mean±SD NON**	**Mean±SD CTLs**
Bacteroidetes	Rikenellaceae			0.0023	CENT- CTLs	0.0034	12.17	↑	0.12±0.18	0.06±0.11	0.07±0.14
Bacteroidetes	Bacteroidaceae	*Bacteroides*	*B. coprocola*	0.0209	CENT- CTLs	0.0333	7.73	↓	0.64±2.45	0.28±0.83	0.98±3.70
Bacteroidetes	Bacteroidaceae	*Bacteroides*	*B. ovatus*	0.0085	CENT- CTLs	0.0179	9.54	↓	0.27±0.51	0.44±0.63	1.27±2.57
Bacteroidetes	Bacteroidaceae	*Bacteroides*	*B. paurosaccharolyticus*	0.0167	CENT- CTLs	0.0176	8.18	↓	0.10±0.13	0.15±0.18	0.18±0.17
Bacteroidetes	Prevotellaceae	*Paraprevotella*	*P. clara*	0.0378	CENT- CTLs	0.0453	6.55	↓	0.04±0.08	0.06±0.11	0.16±0.28
Firmicutes	Synergistaceae			0	CENT- CTLs	0.0067	30.49	↑	0.48±0.88	0.17±0.63	0.03±0.13
Firmicutes	Lachnospiraceae	*Blautia*		0.0376	CENT- CTLs	0.0335	6.56	↓	3.63±2.36	5.48±3.94	6.50±4.86
Firmicutes	Syntrophomonadaceae	Caldicellulosiruptor		0.0102	CENT- CTLs	0.0148	9.17	↑	0.11±0.11	0.08±0.10	0.06±0.07
Firmicutes	Lachnospiraceae	*Blautia*	*B. coccoides*	0.0072	CENT- CTLs	0.0059	9.87	↓	0.63±0.46	1.40±1.31	1.40±1.05
Firmicutes	Erysipelothricaceae	*Erysipelothrix*	*E. inopinata*	0.0317	CENT- CTLs	0.0258	6.9	↓	0.06±0.13	0.13±0.29	0.16±0.49
Proteobacteria	Desulfohalobiaceae			0.014	CENT- CTLs	0.0109	8.53	↑	0.23±0.27	0.11±0.09	0.15±0.27
Proteobacteria	Desulfohalobiaceae	*Desulfonauticus*		0.0142	CENT- CTLs	0.011	8.51	↑	0.23±0.27	0.11±0.09	0.15±0.27
Proteobacteria	Desulfovibrionaceae	*Desulfovibrio*		0.0079	CENT- CTLs	0.0177	9.68	↑	0.44±0.48	0.70±1.66	0.19±0.34
Proteobacteria	Desulfohalobiaceae	*Desulfonauticus*	*D. autotrophicus*	0.0142	CENT- CTLs	0.011	8.51	↑	0.23±0.27	0.11±0.09	0.15±0.27
Synergistetes	Synergistaceae	*Cloacibacillus*		0.0028	CENT- CTLs	0.0018	11.77	↑	0.27±0.71	0.11±0.57	0.02±0.11
Synergistetes	*Synergistaceae*	*Synergistes*		0.0191	CENT- CTLs	0.0148	7.92	↑	0.15±0.45	0.03±0.09	0.01±0.06
Verrucomicrobia				0.0032	CENT- CTLs	0.0036	11.47	↑	10.26±14.88	6.46±10.39	2.20±4.71
Verrucomicrobia	Verrucomicrobiaceae			0.0047	CENT- CTLs	0.0054	10.72	↑	10.19±14.84	6.43±10.36	2.19±4.70
Verrucomicrobia	Verrucomicrobiaceae	*Akkermansia*		0.0054	CENT- CTLs	0.0058	10.45	↑	9.02±13.17	5.67±9.17	1.91±4.12
Verrucomicrobia	Verrucomicrobiaceae	*Luteolibacter*		0.001	CENT- CTLs	0.0012	13.78	↑	0.49±0.70	0.31±0.50	0.11±0.24
Verrucomicrobia	Verrucomicrobiaceae	*Prosthecobacter*		0.0072	CENT- CTLs	0.0098	9.86	↑	0.16±0.22	0.10±0.15	0.04±0.08
Verrucomicrobia	Rubritaleaceae	*Rubritalea*		0.0035	CENT- CTLs	0.0045	11.29	↑	0.38±0.53	0.24±0.39	0.09±0.18
Verrucomicrobia	Verrucomicrobiaceae	*Akkermansia*	*A. muciniphila*	0.0054	CENT- CTLs	0.0058	10.45	↑	9.02±13.16	5.67±9.17	1.91±4.12
Verrucomicrobia	Verrucomicrobiaceae	*Luteolibacter*	*L. algae*	0.001	CENT- CTLs	0.0012	13.78	↑	0.49±0.70	0.31±0.50	0.11±0.24

Table shows the 24 GM significant differences observed only from the comparison between CENT and CTLs, performed by Kruskal-Wallis test on R software v.3.5.2. Pairwise comparison was performed only for significant taxa, followed by Bonferroni correction for multiple comparisons. *p* equal to or less than 0.05 was considered statistically significant. CENT= centenarian subjects, NON= nonagenarian subjects, CTLs= healthy younger controls, ↓= significantly reduced in CENT, ↑= significantly increased in CENT.

Table S6 41 Statistically significant divergences in the relative abundance of bacterial taxa observed only from the comparison between NON and CTLs

					**Post-hoc analysis, Bonferroni method (only for significant bacteria)**						
**Phylum**	**Family**	**Genus**	**Species**	**Kruskal-Wallis *p*-value**	**Pairwise group**	**Pairwise *p*-value**	**Chi square (χ^2)**	**↓/↑**	**Mean±SD CENT**	**Mean±SD NON**	**Mean±SD CTLs**
Actinobacteria	Bifidobacteriaceae			0.0037	NON- CTLs	0.0058	11.2	↑	6.95±10.25	8.01±9.71	2.08±3.03
Actinobacteria	Bifidobacteriaceae	*Bifidobacterium*		0.0039	NON- CTLs	0.0061	11.1	↑	6.91±10.18	7.98±9.67	2.07±3.02
Actinobacteria	Eggerthellaceae	*Slackia*		0.0024	NON- CTLs	0.0028	12.07	↑	0.27±0.29	0.29±0.26	0.17±0.26
Actinobacteria	Bifidobacteriaceae	*Bifodobacterium*	*B. angulatum*	0	NON- CTLs	0	24.71	↑	0.01±0.03	0.18±0.60	0.00±0.01
Actinobacteria	Bifidobacteriaceae	*Bifodobacterium*	*B. asteroides*	0.0006	NON- CTLs	0.0011	14.95	↑	0.11±0.18	0.11±0.14	0.02±0.03
Actinobacteria	Bifidobacteriaceae	*Bifodobacterium*	*B. bifidum*	0.0175	NON- CTLs	0.0418	8.09	↑	0.29±0.48	0.11±0.17	0.04±0.17
Actinobacteria	Bifidobacteriaceae	*Bifodobacterium*	*B. catenulatum*	0.0025	NON- CTLs	0.0031	11.95	↑	0.92±3.03	1.03±2.04	0.12±0.33
Actinobacteria	Bifidobacteriaceae	*Bifodobacterium*	*B. choerinum*	0.0118	NON- CTLs	0.033	8.88	↑	0.14±0.19	0.24±0.33	0.09±0.19
Actinobacteria	Bifidobacteriaceae	*Bifodobacterium*	*B. indicum*	0.0018	NON- CTLs	0.0022	12.68	↑	0.14±0.25	0.23±0.25	0.06±0.10
Actinobacteria	Bifidobacteriaceae	*Bifodobacterium*	*B. kashiwanohense*	0.0011	NON- CTLs	0.0008	13.55	↑	0.12±0.34	0.16±0.29	0.02±0.05
Actinobacteria	Coriobacteriaceae	*Collinsella*	*C. aerofaciens*	0.0122	NON- CTLs	0.0126	8.82	↑	0.36±0.42	0.40±0.48	0.21±0.41
Actinobacteria	Coriobacteriaceae	*Collinsella*	*C. tanakaei*	0.001	NON- CTLs	0.0007	13.77	↑	0.09±0.34	0.29±1.26	0.01±0.02
Bacteroidetes	Bacteroidaceae	*Bacteroides*	*B. caccae*	0.0005	NON- CTLs	0.0003	15.31	↓	0.56±0.76	0.27±0.65	0.90±1.09
Bacteroidetes	Bacteroidaceae	*Bacteroides*	*B. cellulosilyticus*	0.011	NON- CTLs	0.0091	9.02	↓	0.37±0.74	0.19±0.56	0.61±1.22
Bacteroidetes	Bacteroidaceae	*Bacteroides*	*B. denticanum*	0.0154	NON- CTLs	0.0363	8.35	↓	0.13±0.31	0.12±0.23	0.52±1.20
Bacteroidetes	Bacteroidaceae	*Bacteroides*	*B. fragilis*	0.0172	NON- CTLs	0.0316	8.13	↑	0.22±0.30	0.68±1.54	0.24±0.71
Bacteroidetes	Bacteroidaceae	*Bacteroides*	*B. stercorirosoris*	0	NON- CTLs	0	23.17	↓	0.40±0.41	0.22±0.24	0.62±0.52
Bacteroidetes	Bacteroidaceae	*Bacteroides*	*B. uniformis*	0.0043	NON- CTLs	0.0042	10.89	↓	3.05±5.92	1.43±2.30	3.09±3.36
Bacteroidetes	Sphingobacteriaceae	*Sphingobacterium*	*S. shayense*	0.0389	NON- CTLs	0.0421	4.49	↓	0.09±0.10	0.08±0.10	0.18±0.27
Chloroflexi	Caldilineaceae			0.0288	NON- CTLs	0.0267	7.09	↓	0.06±0.04	0.05±0.06	0.12±0.13
Cyanobacteria				0.0093	NON- CTLs	0.0071	9.36	↓	0.62±0.81	0.31±0.45	0.95±1.63
Firmicutes	Eubacteriaceae			0.0179	NON- CTLs	0.0445	8.05	↑	0.13±0.10	0.14±0.12	0.09±0.08
Firmicutes	Lactobacillaceae			0.009	NON- CTLs	0.0235	9.43	↑	0.23±0.47	1.04±3.22	0.13±0.17
Firmicutes	Streptococcaceae			0	NON- CTLs	0	23.55	↑	0.72±0.87	1.80±2.18	0.19±0.26
Firmicutes	Acidaminococcaceae	*Acidaminococcus*		0.0084	NON- CTLs	0.006	9.56	↓	0.36±1.22	0.04±0.13	0.54±1.64
Firmicutes	Lactobacillaceae	*Lactobacillus*		0.0064	NON- CTLs	0.0174	10.12	↑	0.22±0.46	1.00±3.09	0.12±0.16
Firmicutes	Acidaminococcaceae	*Phascolarctobacterium*		0.0197	NON- CTLs	0.0393	7.85	↓	1.84±2.79	0.29±0.44	1.35±2.00
Firmicutes	Streptococcaceae	*Streptococcus*		0	NON- CTLs	0	23.67	↑	0.70±0.87	1.78±2.16	0.19±0.25
Firmicutes	Acidaminococcaceae	*Acidaminococcus*	*A. intestini*	0.001	NON- CTLs	0.0009	13.81	↓	0.02±0.06	0.00±0.00	0.14±0.42
Firmicutes	Acidaminococcaceae	*Phascolarctobacterium*	*P. faecium*	0.0048	NON- CTLs	0.0252	10.67	↓	0.68±1.10	0.04±0.12	0.45±1.03
Firmicutes	Streptococcaceae	*Streptococcus*	*S. bovis*	0	NON- CTLs	0	20.22	↑	0.03±0.03	0.24±0.55	0.03±0.13
Firmicutes	Streptococcaceae	*Streptococcus*	*S. parasanguinis*	0	NON- CTLs	0	24.27	↑	0.05±0.08	0.19±0.30	0.01±0.01
Firmicutes	Streptococcaceae	*Streptococcus*	*S. vestibularis*	0.0066	NON- CTLs	0.0046	10.05	↑	0.18±0.31	0.57±0.89	0.05±0.10
Firmicutes	Veillonellaceae	*Veillonella*	*V. atypica*	0.0122	NON- CTLs	0.0091	8.82	↑	0.05±0.14	0.12±0.22	0.04±0.11
Firmicutes	Veillonellaceae	*Veillonella*	*V. dispar*	0.0195	NON- CTLs	0.0155	7.87	↑	0.05±0.18	0.11±0.28	0.02±0.04
Proteobacteria	Enterobacteriaceae	*Enterobacter*		0.0246	NON- CTLs	0.0201	7.41	↑	0.34±0.67	0.76±2.35	0.11±0.35
Proteobacteria	Enterobacteriaceae	*Escherichia*		0.0022	NON- CTLs	0.0019	12.28	↑	7.00±12.18	3.14±6.70	0.22±0.65
Proteobacteria	Yersiniaceae	*Serratia*		0.0007	NON- CTLs	0.0005	14.56	↑	1.15±1.92	0.67±1.10	0.06±0.13
Proteobacteria	Zoogloeaceae	*Uliginosibacterium*		0.0327	NON- CTLs	0.0356	6.84	↓	0.02±0.07	0.00±0.00	0.20±1.25
Proteobacteria	Enterobacteriaceae	*Candidatus Blochmannia*	*C. B. rufipes*	0.0118	NON- CTLs	0.0123	8.89	↓	0.00±0.00	0.00±0.00	0.65±0.76
Proteobacteria	Enterobacteriaceae	*Escherichia*	*E. albertii*	0.004	NON- CTLs	0.0037	11.06	↑	5.67±9.94	2.54±5.38	0.20±0.60
Proteobacteria	*Yersiniaceae*	*Serratia*	*S. entomophila*	0.0009	NON- CTLs	0.0007	14.12	↑	1.13±1.90	0.66±1.09	0.06±0.13

Table shows the 41 GM significant differences observed only from the comparison between NON and CTLs, performed by Kruskal-Wallis test on R software v.3.5.2. Pairwise comparison was performed only for significant taxa, followed by Bonferroni correction for multiple comparisons. *p* equal to or less than 0.05 was considered statistically significant. CENT= centenarian subjects, NON= nonagenarian subjects, CTLs= healthy younger controls, ↓= significantly reduced in NON, ↑= significantly increased in NON.

Table S7 8 Statistically significant divergences in the relative abundance of bacterial taxa observed from the comparison between CENT and NON

					Post-hoc analysis, Bonferroni method (only for significant bacteria)						
**Phylum**	**Family**	**Genus**	**Species**	**Kruskal-Wallis *p*-value**	**Pairwise group**	**Pairwise *p*-value**	**Chi square (χ^2)**	**↓/↑**	**Mean±SD CENT**	**Mean±SD NON**	**Mean±SD CTLs**
Actinobacteria	Bifidobacteriaceae	*Bifodobacterium*	*B. angulatum*	0	CENT- NON	0.0035	24.71	↓	0.01±0.03	0.18±0.60	0.00±0.01
Actinobacteria	Bifidobacteriaceae	*Bifodobacterium*	*B. catenulatum*	0.0025	CENT- NON	0.0347	11.95	↓	0.92±3.03	1.03±2.04	0.12±0.33
Bacteroidetes	Bacteroidaceae	*Bacteroides*	*B. intestinalis*	0.0184	CENT- NON	0.0202	7.99	↑	0.22±0.53	0.00±0.01	0.11±0.54
Firmicutes	Lactobacillaceae			0.009	CENT- NON	0.0267	9.43	↓	0.23±0.47	1.04±3.22	0.13±0.17
Firmicutes	Eubacteriaceae	*Eubacterium*		0.0296	CENT- NON	0.0283	7.04	↓	0.13±0.21	0.30±0.82	0.05±0.10
Firmicutes	Lactobacillaceae	*Lactobacillus*		0.0064	CENT- NON	0.0204	10.12	↓	0.22±0.46	1.00±3.09	0.12±0.16
Firmicutes	Lachnospiraceae	*Blautia*	*B. coccoides*	0.0072	CENT- NON	0.0375	9.87	↓	0.63±0.46	1.40±1.31	1.40±1.05
Firmicutes	Acidaminococcaceae	*Phascolarctobacterium*	*P. faecium*	0.0048	CENT- NON	0.0095	10.67	↑	0.68±1.10	0.04±0.12	0.45±1.03

Table shows the 8 GM significant differences observed from the comparison between CENT and NON, performed by Kruskal-Wallis test on R software v.3.5.2. Pairwise comparison was performed only for significant taxa, followed by Bonferroni correction for multiple comparisons. *p* equal to or less than 0.05 was considered statistically significant. CENT= centenarian subjects, NON= nonagenarian subjects, CTLs= healthy younger controls, ↓= significantly reduced in CENT, ↑= significantly increased in CENT.

Table S8 Spearman's correlation between significant taxa and dietary, lifestyle, and clinical parameters in CENT

**Phylum**	**Family**	***Genus***	***Species***	**Variable**	**R**	***p*-value**
Fusobacteria				Age	0.612	0.009
Fusobacteria	Fusobacteriaceae			Age	0.659	0.004
Bacteroidetes	Bacteroidaceae	*Bacteroides*	*B. coprocola*	Age	-0.489	0.046
Cyanobacteria				BMI	0.581	0.018
Cyanobacteria	Nostocaceae			BMI	0.54	0.031
Proteobacteria	Zoogloeaceae	*Uliginosibacterium*		BMI	-0.503	0.047
Bacteroidetes	Prevotellaceae	*Prevotella*	*P. shahii*	BMI	-0.56	0.024
Firmicutes	Erysipelothricaceae	*Erysipelothrix*	*E. inopinata*	Alcohol_consumption	0.495	0.043
Firmicutes	Thermicanaceae			Alcohol_consumption	0.529	0.029
Firmicutes	Thermicanaceae	*Thermicanus*		Alcohol_consumption	0.529	0.029
Firmicutes	Thermicanaceae			Former_Alcohol_consumption	0.505	0.039
Firmicutes	Thermicanaceae	*Thermicanus*		Former_Alcohol_consumption	0.505	0.039
Proteobacteria	Desulfovibrionaceae	*Desulfovibrio*		Former_Alcohol_consumption	-0.515	0.034
Actinobacteria	Eggerthellaceae	*Eggerthella*		Coffee_consumption	0.596	0.012
Euryarchaeota				Coffee_consumption	0.488	0.047
Euryarchaeota	Methanobacteriaceae			Coffee_consumption	0.488	0.047
Euryarchaeota	Methanobacteriaceae	*Methanobrevibacter*		Coffee_consumption	0.488	0.047
Euryarchaeota	Methanobacteriaceae	*Methanobrevibacter*	*M. smithii*	Coffee_consumption	0.488	0.047
Firmicutes	Erysipelothricaceae	*Erysipelothrix*	*E. inopinata*	Bowel_function	0.5	0.041
Firmicutes	Lactobacillaceae	*Lactobacillus*	*L. taiwanensis*	Bowel_function	-0.645	0.005
Firmicutes	Thermicanaceae			Bowel_function	0.506	0.038
Firmicutes	Thermicanaceae	*Thermicanus*		Bowel_function	0.506	0.038
Proteobacteria	Desulfohalobiaceae			Bowel_function	0.652	0.005
Proteobacteria	Desulfohalobiaceae	*Desulfonauticus*		Bowel_function	0.652	0.005
Proteobacteria	Desulfohalobiaceae	*Desulfonauticus*	*D. autotrophicus*	Bowel_function	0.652	0.005
Synergistetes				Bowel_function	0.571	0.017
Synergistetes	Synergistaceae			Bowel_function	0.563	0.019
Firmicutes	Coprobacillaceae			Number_medications	-0.542	0.025
Synergistetes	Synergistaceae	*Cloacibacillus*		Number_medications	-0.483	0.05
Actinobacteria	Bifidobacteriaceae	*Bifidobacterium*	*B. angulatum*	Number_medications	0.573	0.016

Actinobacteria	Coriobacteriaceae	*Collinsella*	*C. aerofaciens*	Number_medications	-0.587	0.013
Firmicutes	Streptococcaceae			ADL_score	-0.534	0.027
Proteobacteria	Desulfovibrionaceae	*Desulfovibrio*	*D. piger*	ADL_score	-0.626	0.007
Actinobacteria	Eggerthellaceae	*Eggerthella*		DMS_score	0.536	0.027
Bacteroidetes	Bacteroidaceae	*Bacteroides*	*B. rodentium*	DMS_score	-0.516	0.034
Bacteroidetes	Tannerellaceae	*Parabacteroides*	*P. merdae*	DMS_score	-0.778	<.001
Firmicutes	Lactobacillaceae	*Lactobacillus*	*L. taiwanensis*	DMS_score	-0.671	0.003
Firmicutes	Clostridiaceae			DMS_score	0.565	0.018
Firmicutes	Clostridiaceae	*Clostridium*		DMS_score	0.593	0.012
Firmicutes	Clostridiaceae	*Dorea*		DMS_score	0.494	0.044
Firmicutes	Peptostreptococcaceae	*Peptoniphilus*		DMS_score	0.591	0.012
Firmicutes	Thermicanaceae			DMS_score	0.548	0.023
Firmicutes	Thermicanaceae	*Thermicanus*		DMS_score	0.548	0.023
Firmicutes	Erysipelotrichidae	*Catenibacterium*		DMS_score	-0.544	0.024
Firmicutes	Acidaminococcaceae	*Veillonella*		DMS_score	-0.482	0.05
Firmicutes	Acidaminococcaceae	*Dialister invisus*		DMS_score	-0.484	0.049
Proteobacteria	Desulfovibrionaceae	*Desulfovibrio*		MMSE_score	-0.552	0.033
Proteobacteria	Desulfovibrionaceae	*Desulfovibrio*		MNA_score	-0.665	0.004
Proteobacteria	Desulfovibrionaceae	*Desulfovibrio*	*D. piger*	MNA_score	-0.643	0.005
Proteobacteria	Desulfovibrionaceae	*Desulfovibrio*	*D. piger*	PASE_score	-0.502	0.04

Spearman's correlation was calculated in SPSS software v.28.0.1.0. Only significant bacterial taxa identified in the univariate analysis were correlated to clinical parameters. BMI= Body Mass Index, MMSE= Mini Mental State Evaluation, MDS= Mediterranean Diet score, ADL= Activities of Daily Living, PASE= Physical Activity Scale for the Elderly, MNA= Mini Nutritional Assessment. R = r coefficient; *p*-value equal to or less than 0.05 was considered statistically significant.

Table S9 Spearman's correlation between significant taxa and dietary, lifestyle, and clinical parameters in NON

**Phylum**	**Family**	***Genus***	***Species***	**Variable**	**R**	***p*-value**
Actinobacteria	Eggerthellaceae	*Slackia*		Age	-0.384	0.04
Firmicutes	Streptococcaceae			BMI	0.548	0.01
Firmicutes	Streptococcaceae	*Streptococcus*		BMI	0.552	0.009
Firmicutes	Streptococcaceae	*Streptococcus*	*S. bovis*	BMI	0.571	0.007
Actinobacteria	Bifidobacteriaceae			Alcohol_consumption	-0.386	0.038
Actinobacteria	Bifidobacteriaceae	*Bifidobacterium*		Alcohol_consumption	-0.396	0.033
Actinobacteria	Bifidobacteriaceae	*Bifidobacterium*	*B. angulatum*	Alcohol_consumption	-0.43	0.02
Actinobacteria	Bifidobacteriaceae	*Bifidobacterium*	*B. catenulatum*	Alcohol_consumption	-0.431	0.02
Actinobacteria	Bifidobacteriaceae	*Bifidobacterium*	*B. indicum*	Alcohol_consumption	-0.425	0.022
Actinobacteria	Bifidobacteriaceae	*Bifidobacterium*	*B. kashiwanohense*	Alcohol_consumption	-0.396	0.033
Actinobacteria	Coriobacteriaceae			Alcohol_consumption	-0.51	0.005
Actinobacteria	Coriobacteriaceae	*Collinsella*		Alcohol_consumption	-0.452	0.014
Bacteroidetes	Bacteroidaceae	*Bacteroides*	*B. denticanum*	Former_Alcohol_consumption	0.469	0.01
Euryarcheota				Former_Alcohol_consumption	0.387	0.038
Euryarcheota	Methanobacteriaceae			Former_Alcohol_consumption	0.387	0.038
Euryarcheota	Methanobacteriaceae	*Methanobrevibacter*		Former_Alcohol_consumption	0.435	0.018
Euryarcheota	Methanobacteriaceae	*Methanobrevibacter*	*M. smithii*	Former_Alcohol_consumption	0.43	0.02
Proteobacteria	Xanthomonadaceae			Former_Alcohol_consumption	-0.463	0.011
Bacteroidetes	Bacteroidaceae	*Bacteroides*	*B. uniformis*	Coffe_consumption	-0.384	0.039
Actinobacteria	Bifidobacteriaceae	*Bifidobacterium*	*B. angulatum*	Bowel_function	0.399	0.032
Actinobacteria	Bifidobacteriaceae	*Bifidobacterium*	*B. bifidum*	Bowel_function	0.41	0.027
Euryarcheota				Bowel_function	-0.389	0.037
Euryarcheota	Methanobacteriaceae			Bowel_function	-0.389	0.037
Firmicutes	Acidaminococcaceae	*Phascolarctobacterium*	*P. faecium*	Bowel_function	0.384	0.04
Actinobacteria	Bifidobacteriaceae			Number_medications	-0.414	0.036
Actinobacteria	Bifidobacteriaceae	*Bifidobacterium*		Number_medications	-0.423	0.001
Actinobacteria	Bifidobacteriaceae	*Bifidobacterium*	*B. choerinum*	Number_medications	-0.51	0.008
Actinobacteria	Bifidobacteriaceae	*Bifidobacterium*	*B. indicum*	Number_medications	-0.431	0.028
Bacteroidetes				Number_medications	0.616	0.001

Bacteroidetes	Bacteroidaceae			Number_medications	-0.414	0.036
Bacteroidetes	Bacteroidaceae	*Bacteroides*		Number_medications	0.579	0.002
Bacteroidetes	Bacteroidaceae	*Bacteroides*	*B. rodentium*	Number_medications	0.483	0.012
Bacteroidetes	Bacteroidaceae	*Bacteroides*	*B. sartorii*	Number_medications	0.714	0.0000422
Bacteroidetes	Bacteroidaceae	*Bacteroides*	*B. uniformis*	Number_medications	0.468	0.016
Firmicutes	Sphingobacteriaceae	*Sphingobacterium*	*S. shayense*	Number_medications	0.442	0.024
Firmicutes	Lachnospiraceae	*Blautia*	*B. wexlerae*	Number_medications	-0.446	0.022
Proteobacteria	Alcaligenaceae			Number_medications	0.47	0.015
Proteobacteria	Sutterellaceae	*Sutterella*		Number_medications	0.47	0.015
Proteobacteria	Desulfovibrionaceae	*Desulfovibrio*	*D. fairfieldensis*	ADL_score	-0.399	0.032
Proteobacteria	Enterobacteriaceae	*Enterobacter*	*E. soli*	ADL_score	0.424	0.022
Proteobacteria	Enterobacteriaceae	*Escherichia*		ADL_score	-0.4	0.032
Proteobacteria	Enterobacteriaceae	*Escherichia*	*E. albertii*	ADL_score	-0.397	0.033
Proteobacteria	Yersiniaceae	*Serratia*		ADL_score	-0.393	0.035
Proteobacteria	Yersiniaceae	*Serratia*	*S. entomophila*	ADL_score	-0.393	0.035
Actinobacteria				DMS_score	-0.46	0.012
Actinobacteria	Bififobacteriaceae			DMS_score	-0.466	0.011
Actinobacteria	Bififobacteriaceae	*Bifidobacterium*		DMS_score	-0.47	0.01
Actinobacteria	Bififobacteriaceae	*Bifidobacterium*	*B. adolescentis*	DMS_score	-0.509	0.005
Actinobacteria	Bififobacteriaceae	*Bifidobacterium*	*B. angulatum*	DMS_score	-0.465	0.011
Actinobacteria	Bififobacteriaceae	*Bifidobacterium*	*B. asteroides*	DMS_score	-0.466	0.011
Actinobacteria	Bifidobacteriaceae	*Bifidobacterium*	*B. bifidum*	DMS_score	-0.398	0.032
Actinobacteria	Bifidobacteriaceae	*Bifidobacterium*	*B. catenulatum*	DMS_score	-0.405	0.029
Actinobacteria	Bififobacteriaceae	*Bifidobacterium*	*B. indicum*	DMS_score	-0.346	0.066
Actinobacteria	Bififobacteriaceae	*Bifidobacterium*	*B. kashiwanohense*	DMS_score	-0.44	0.017
Actinobacteria	Bififobacteriaceae	*Bifidobacterium*	*B. stercoris*	DMS_score	-0.431	0.02
Actinobacteria	Streptomycetaceae			DMS_score	-0.406	0.029
Bacteroidetes	Bacteroidaceae	*Bacteroides*	*B. clarus*	DMS_score	-0.431	0.02
Bacteroidetes	Bacteroidaceae	*Bacteroides*	*B. dorei*	DMS_score	0.568	0.001
Bacteroidetes	Odoribacteraceae			DMS_score	0.376	0.044
Bacteroidetes	Sphingobacteriaceae	*Pedobacter*	*P. kwangyangensis*	DMS_score	0.387	0.038
Bacteroidetes	Tannerellaceae	*Parabacteroides*	*P. goldsteinii*	DMS_score	0.399	0.032
Bacteroidetes	Tannerellaceae	*Parabacteroides*	*P. johnsonii*	DMS_score	0.514	0.004
Firmicutes	Clostridiaceae	*Alkaliphilus*		DMS_score	0.415	0.025
Firmicutes	Clostridiaceae	*Clostridium*	*C. frigoris*	DMS_score	0.374	0.045
Firmicutes	Lactobacillaceae	*Lactobacillus*	*L. ultunensis*	DMS_score	-0.395	0.034
Firmicutes	Peptostreptococcaceae	*Peptoniphilus*		DMS_score	0.381	0.041
Proteobacteria	Desulfovibrionaceae	*Bilophila*		DMS_score	0.381	0.042
Proteobacteria	Desulfovibrionaceae	*Bilophila*	*B. wadsworthia*	DMS_score	0.374	0.046
Bacteroidetes				PASE_score	-0.512	0.005
Bacteroidetes	Bacteroidaceae	*Bacteroides*	*B. cellulosilyticus*	PASE_score	-0.388	0.038
Bacteroidetes	Bacteroidaceae	*Bacteroides*	*B. uniformis*	PASE_score	-0.379	0.042
Bacteroidetes	Bacteroidaceae	*Bacteroides*	*B. xylanisolvens*	PASE_score	-0.401	0.031
Firmicutes	Thermicanaceae			PASE_score	0.466	0.011
Firmicutes	Thermicanaceae	*Thermicanus*		PASE_score	0.466	0.011
Firmicutes	Lachnospiraceae	*Blautia*	*B. wexlerae*	PASE_score	0.382	0.041
Proteobacteria	Alcaligenaceae			PASE_score	-0.384	0.04
Proteobacteria	Sutterellaceae	*Sutterella*		PASE_score	-0.4	0.032

Spearman's correlation was calculated in SPSS software (v.28.0.1.0). Only significant bacterial taxa identified in the univariate analysis were correlated to clinical parameters. BMI= Body Mass Index, MMSE= Mini Mental State Evaluation, MDS= Mediterranean Diet score, ADL= Activities of Daily Living, PASE= Physical Activity Scale for the Elderly, MNA= Mini Nutritional Assessment. R= r coefficient; *p*-value equal to or less than 0.05 was considered statistically significant.

References

1. Panagiotakos, D. B., Pitsavos, C., Arvaniti, F., & Stefanadis, C. (2007). Adherence to the Mediterranean food pattern predicts the prevalence of hypertension, hypercholesterolemia, diabetes and obesity, among healthy adults; the accuracy of the MedDietScore. *Preventive medicine*, *44*(4), 335–340. https://doi.org/10.1016/j.ypmed.2006.12.009
2. Folstein, M. F., Folstein, S. E., & McHugh, P. R. (1975). "Mini-mental state". A practical method for grading the cognitive state of patients for the clinician. *Journal of psychiatric research*, *12*(3), 189–198. https://doi.org/10.1016/0022-3956(75)90026-6
3. Crum, R. M., Anthony, J. C., Bassett, S. S., & Folstein, M. F. (1993). Population-based norms for the Mini-Mental State Examination by age and educational level. *JAMA*, *269*(18), 2386–2391.
4. Katz, S., Downs, T. D., Cash, H. R., & Grotz, R. C. (1970). Progress in development of the index of ADL. *The Gerontologist*, *10*(1), 20–30. https://doi.org/10.1093/geront/10.1_part_1.20
5. Guigoz, Y., Lauque, S., & Vellas, B. J. (2002). Identifying the elderly at risk for malnutrition. The Mini Nutritional Assessment. *Clinics in geriatric medicine*, *18*(4), 737–757. https://doi.org/10.1016/s0749-0690(02)00059-9
6. Valentini, A., Federici, M., Cianfarani, M. A., Tarantino, U., & Bertoli, A. (2018). Frailty and nutritional status in older people: the Mini Nutritional Assessment as a screening tool for the identification of frail subjects. *Clinical interventions in aging*, *13*, 1237–1244. https://doi.org/10.2147/CIA.S164174
7. Washburn, R. A., Smith, K. W., Jette, A. M., & Janney, C. A. (1993). The Physical Activity Scale for the Elderly (PASE): development and evaluation. *Journal of clinical epidemiology*, *46*(2), 153–162. https://doi.org/10.1016/0895-4356(93)90053-4
